# Electrocardiogram-less, free-breathing myocardial extracellular volume fraction mapping in small animals at high heart rates using motion-resolved cardiovascular magnetic reesonance multitasking: a feasibility study in a heart failure with preserved ejection fraction rat model

**DOI:** 10.1186/s12968-020-00699-9

**Published:** 2021-02-11

**Authors:** Pei Han, Rui Zhang, Shawn Wagner, Yibin Xie, Eugenio Cingolani, Eduardo Marban, Anthony G. Christodoulou, Debiao Li

**Affiliations:** 1 Department of Bioengineering, University of California, Los Angeles, Los Angeles, CA USA; 2 Biomedical Imaging Research Institute, Cedars-Sinai Medical Center, Los Angeles, CA USA; 3 Smidt Heart Institute, Cedars-Sinai Medical Center, Los Angeles, CA USA; 4 Department of Cardiology, Xinhua Hospital Affiliated to Shanghai Jiaotong University School of Medicine, Shanghai, China

**Keywords:** Cardiovascular MR, T_1_ mapping, Extracellular volume fraction, HFpEF

## Abstract

**Background:**

Extracellular volume fraction (ECV) quantification with cardiovascular magnetic resonance (CMR) T_1_ mapping is a powerful tool for the characterization of focal or diffuse myocardial fibrosis. However, it is technically challenging to acquire high-quality T_1_ and ECV maps in small animals for preclinical research because of high heart rates and high respiration rates. In this work, we developed an electrocardiogram (ECG)-less, free-breathing ECV mapping method using motion-resolved CMR Multitasking on a 9.4 T small animal CMR system. The feasibility of characterizing diffuse myocardial fibrosis was tested in a rat heart failure model with preserved ejection fraction (HFpEF).

**Methods:**

High-salt fed rats diagnosed with HFpEF (n = 9) and control rats (n = 9) were imaged with the proposed ECV Multitasking technique. A 25-min exam, including two 4-min T_1_ Multitasking scans before and after gadolinium injection, were performed on each rat. It allows a cardiac temporal resolution of 20 ms for a heart rate of ~ 300 bpm. Myocardial ECV was calculated from the hematocrit (HCT) and fitted T_1_ values of the myocardium and the blood pool. Masson's trichrome stain was used to measure the extent of fibrosis. Welch’s t-test was performed between control and HFpEF groups.

**Results:**

ECV was significantly higher in the HFpEF group (22.4% ± 2.5% vs. 18.0% ± 2.1%, *P* = 0.0010). A moderate correlation between the ECV and the extent of fibrosis was found (*R* = 0.59, *P* = 0.0098).

**Conclusions:**

Motion-resolved ECV Multitasking CMR can quantify ECV in the rat myocardium at high heart rates without ECG triggering or respiratory gating. Elevated ECV found in the HFpEF group is consistent with previous human studies and well correlated with histological data. This technique has the potential to be a viable imaging tool for myocardial tissue characterization in small animal models.

## Background

Cardiovascular magnetic resonance (CMR) T_1_ mapping is a powerful diagnostic modality for various abnormalities of the myocardium, such as edema, amyloidosis, and overload of lipid or iron [1–3]. Combined with gadolinium (Gd) contrast enhancement, T_1_ mapping allows extracellular volume fraction (ECV) quantification, which can be used to characterize focal or diffuse myocardial fibrosis [3–6].

Rodent models are widely used in preclinical studies of myocardial diseases because of the short development period, availability of genetically modified disease models, and low cost. However, it is technically challenging to acquire high-quality T_1_ and ECV maps in small animals because of their high heart rates (often > 300 bpm) and high respiration rates (around 60 cpm). Therefore, CMR quantification of ECV is still not well established despite the important unmet needs in research. Several studies have been done to improve CMR T_1_ mapping and/or ECV measurement in small animals. Coolen et al. proposed a 3D T_1_ mapping method of the mouse heart using variable flip angle (VFA) analysis [7]. The method has the ability to detect regional differences in myocardium with excellent repeatability, but VFA-based methods have inherent problems with B_1_ inhomogeneity, and the long scan time (more than 20 min) makes it impractical to be used in pre- and post-Gd studies. Messroghli et al. acquired myocardial T_1_ and ECV maps in rats from a single (unsegmented) dataset using a small animal Look-Locker inversion recovery (SALLI) method [8, 9]. It was able to reconstruct both cine CMR images and T_1_ maps, and showed the feasibility to detect diffuse myocardial fibrosis, while the spatial resolution was limited due to signal-to-noise ratio (SNR) consideration. Segmented multi-shot FLASH methods were then proposed to acquired images with higher resolution [10, 11].

In all previous studies with a Look-Locker scheme [12, 13], electrocardiogram (ECG) triggering was used to monitor cardiac motion [8–11]. However, ECG triggering at high field strengths is unreliable due to elevated magnetohydrodynamic effects, which can introduce trigger-related motion artifacts and blurring effects [14, 15]. High heart rates may make the situation even worse. Respiratory navigation was used in some studies [10], however, most studies simply used signal averaging, resulting in image blurring [8, 11]. Furthermore, ECG and respiratory gating setup leads to complicated workflow.

In this study, we developed a ECG-less, free-breathing ECV mapping method using CMR Multitasking [16, 17] (hereinafter abbreviated as *ECV Multitasking*) on a 9.4 T small animal CMR system. It allows continuous acquisition without ECG triggering or respiratory gating. The pre- and post-Gd data were acquired separately but reconstructed jointly, to allow image co-registration and direct ECV mapping. The feasibility of characterizing diffuse myocardial fibrosis was tested in a rat hypertensive heart failure model with preserved ejection fraction (HFpEF), which has been shown to have increased left ventricular (LV) interstitial fibrosis [18–20], and has been recapitulated in recent studies [21–23].

## Methods

### Animal model

All animal experiments were approved by the Cedars-Sinai Institutional Animal Care and Use Committee. Dahl salt-sensitive (DSS) rats can develop hypertension followed by HFpEF on a high-salt diet [18, 19]. In this model, male DSS rats (Charles River Laboratories, Wilmington, Massachusetts, USA) were normally fed (0.3% NaCl) until the age of 7 weeks. Rats were then randomly assigned to a high-salt (HS) diet group (8% NaCl) to induce HFpEF or a normal-salt (NS) diet group (0.3% NaCl) to serve as controls, until the age of 14 weeks [24]. HS rats with heart failure symptoms (including decreased activity, cachexia, labored breathing, and body edema) and echocardiographic evidence for diastolic dysfunctionwere diagnosed as HFpEF.

Control rats (n = 9; weight, 335 ± 44 g) and HS fed rats diagnosed with HFpEF (n = 9; weight, 286 ± 47 g) were imaged. Imaging experiments and all measurements were done between the age of 14 weeks and 15 weeks. After the imaging study, animals were euthanized, and the hearts were excised. Mid-ventricular heart tissues of the control rats (n = 9) and HFpEF rats (n = 9) were sectioned and stained with Masson’s trichrome staining.

### CMR protocol

All CMR data were acquired on a 9.4 T preclinical system (BioSpec 94/20 USR; Bruker Biospin, Billerica, Massachusetts, USA) using a single-channel volume coil. The T_1_ Multitasking sequence was implemented in Paravision 5.1 by modifying the built-in FLASH sequence.

The rat was anesthetized before the scan, and the tail vein was cannulated for later Gd injection within the scan. During the scan, anesthesia was maintained by ventilation with 1.5% isoflurane-oxygen. After the scan, hematocrit (HCT) level was measured for ECV calculation.

**Fig. 1:**
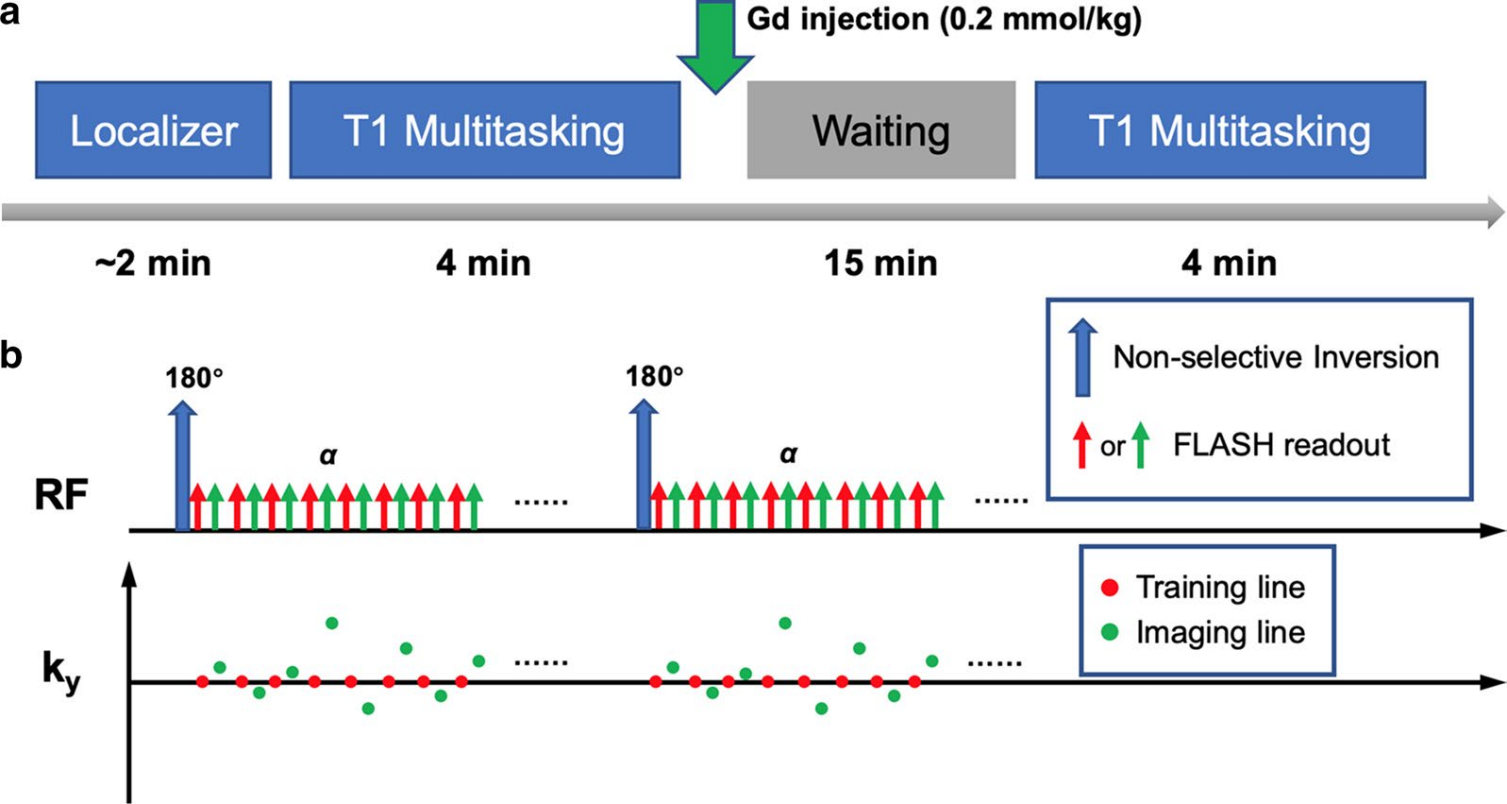
Imaging workflow and T_1_ Multitasking sequence diagram. **a** Imaging workflow: A self-gated (IntraGate) localizer was used to select a mid-cavity short-axis slice of the left ventricle (LV). The CMR Multitasking T_1_ mapping sequence (T_1_ Multitasking) was then performed. Gd contrast agent (Gadavist, 0.2 mmol/kg) was manually administered. 15 min after injection, T_1_ Multitasking was repeated. The total exam time was around 25 min. **b **Sequence design: T_1_ Multitasking was performed using a continuous FLASH acquisition with repeated non-selective inversion recovery (IR) magnetization preparation pulses. Odd-numbered readouts followed randomized Gaussian-density sampling in the phase encoding (${k}_{y}$) direction (used as the imaging data), and even-numbered readouts collected the k-space center line (${k}_{y}=0$, used as the subspace training data)

Figure 1a shows the imaging workflow for one rat study. A self-gated (IntraGate) localizer was used to select a slice with a mid-cavity short-axis LV view. The T_1_ Multitasking sequence [17] (Fig. 1b) was then performed. Gd contrast agent (Gadavist, 0.2 mmol/kg; Bayer Schering Pharma, Berlin-Wedding, Germany) was manually injected immediately afterwards. Fifteen minutes later, the T_1_ Multitasking sequence was repeated on the same slice using identical imaging parameters. The total exam time was ~ 25 min.

Imaging parameters were: matrix size = 128 × 128, FOV = 40 × 40 mm^2^, voxel size = 0.31 × 0.31 × 1.5 mm^3^, flip angle = 5°, TR/TE = 7.0/2.4 ms, recovery period (time between adjacent inversion recovery (IR) pulses) = 2.9 s. In each T_1_ Multitasking module, 85 IR preparation pulses were applied, resulting in a total scan time of 4 min 10 s.

Data acquired from pre- and post-Gd T_1_ Multitasking sequence were reconstructed and analyzed jointly, as described in the following sections. The joint pre- and post-Gd reconstruction can improve respiratory and cardiac binning and promote image co-registration in native and post-Gd T_1_ fitting.

### Image reconstruction

Images from the proposed ECV Multitasking protocol are represented as a high-dimensional image $A(\mathbf{x},{t}_{c},{t}_{r},{t}_{\text{T}_{1}})$ with 2 spatial dimensions and 3 temporal dimensions (cardiac phase $t_{c}$, respiratory phase ${t}_{r}$, and T_1_ recovery time ${t}_{\text{T}_{1}}$). The high-dimensional image can be discretized and viewed as a low-rank tensor $\mathcal{A}$, and thus partially separable [25], i.e.

1 $${\mathbf{A}}_{\left( 1 \right)} = {\mathbf{U}}_{{\mathbf{x}}} {\mathbf{C}}_{\left( 1 \right)} \left( {{\mathbf{U}}_{{\text{c}}} \otimes {\mathbf{U}}_{{\text{r}}} \otimes {\mathbf{U}}_{{{\text{T}}_{1} }} } \right)^{{\text{T}}}$$

where columns of ${\mathbf{U}}_{\mathbf{x}}$ represents spatial basis functions; columns of ${\mathbf{U}}_{{\mathbf{c}}}$, ${\mathbf{U}}_{{\mathbf{r}}}$, and ${\mathbf{U}}_{{{\text{T}}_{1} }}$ contain cardiac, respiratory, and T_1_ recovery temporal basis functions, respectively; $\otimes$ denotes the Kronecker product; and ${\mathbf{A}}_{\left(1\right)}$ and ${\mathbf{C}}_{\left(1\right)}$ are mode-1 matricization of the image tensor $\mathcal{A}$ and the core tensor $\mathcal{C}$ respectively [26].

Equation (1) permits ${\mathbf{A}}_{\left(1\right)}={\mathbf{U}}_{\mathbf{x}}{\varvec{\Phi}}$, where ${\varvec{\Phi}}={\mathbf{C}}_{\left(1\right)}{\left({\mathbf{U}}_{\text{c}}\otimes {\mathbf{U}}_{\text{r}}\otimes {\mathbf{U}}_{{\text{T}}_{1}}\right)}^{\text{T}}$, in which ${\varvec{\Phi}}$ and ${\mathbf{U}}_{\mathbf{x}}$ contain separate temporal and spatial bases respectively. Therefore, ${\varvec{\Phi}}$ can be first recovered using only the training data ${\mathbf{d}}_{\text{tr}}$, which is frequently sampled in time, via Bloch-constrained low-rank tensor completion followed by high-order singular value decomposition (HOSVD) [16, 27]. With ${\varvec{\Phi}}$ determined, the spatial basis ${\mathbf{U}}_{\mathbf{x}}$ can then be reconstructed from the imaging data ${\mathbf{d}}_{\text{im}}$ by solving the following problem:

2 $${\hat{\mathbf{U}}}_{{\mathbf{x}}} = \arg\min_{{\mathbf{U_x}}} \|{\mathbf{d}}_{{{\text{im}}}} - {\Omega }\left( {{\mathbf{EU}}_{{\mathbf{x}}} {{\varvec{\Phi}}}} \right)\|_{2}^{2} + \lambda R\left( {{\mathbf{U}}_{{\mathbf{x}}} } \right)$$

where $\mathbf{E}$ is the signal encoding operator, including Fourier transformation and coil sensitivities weighting (optional), $\Omega$ is the undersampling operator, and $R$ is a regularization functional which was chosen here as a wavelet sparsity penalty in order to additionally exploit compressed sensing [28].

A detailed description of the basic image model and reconstruction scheme used in CMR Multitasking can be found in previous work [16, 17, 29]. In this work, it is divided into the following steps:

#### Determination of T_1_ recovery basis functions in ${\text{U}}_{{\text{T}}_{1}}$ with Bloch simulation

**Fig. 2:**
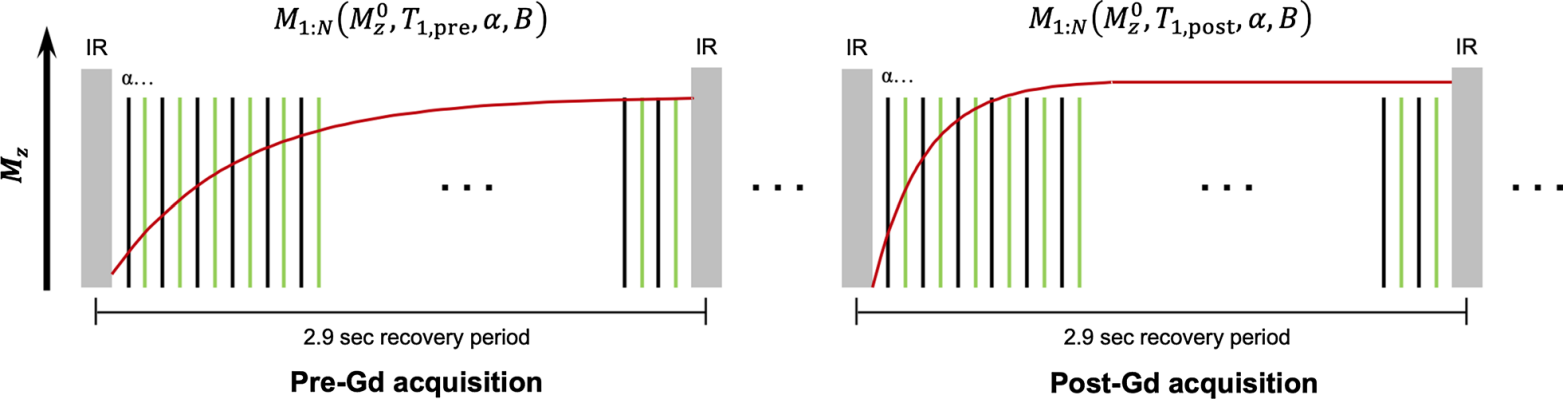
Illustration of joint pre- and post-Gd T_1_ recovery modeling. Representative T_1_ recovery curves from pre- and post-Gd acquisition are shown separately. Black line readouts represent image data, while green line readouts represent training data. A pair of single blocks from pre- and post-Gd acquisition are Bloch simulated together, with the same FLASH flip angle $\alpha$ and inversion efficiency $B$

The inversion recovery (IR)-prepared T_1_ recovery process is modeled by Bloch simulations to determine ${\mathbf{U}}_{{\text{T}}_{1}}$. In this work, pairs of pre- and post-Gd T_1_ recovery curves were jointly simulated as shown in Fig. 2, for the purposes of joint physical modeling during respiratory and cardiac binning (Step 3) and multidimensional image reconstruction (Step 4). The detailed information of the T_1_ recovery modeling is available in Appendix.

A dictionary was generated with this model using 101 T_1_ values ranging from 100 to 3000 ms (${T}_{1,\text{pre}}>{T}_{1,\text{post}}$ combinations only), 15 FLASH flip angles from 0.5 to 7.5$^\circ$, and 21 inversion efficiency values from -1 (complete inversion) to 0 (no inversion). The singular value decomposition (SVD) of this dictionary yielded the T_1_ recovery basis functions ${\mathbf{U}}_{{\text{T}}_{1}}$.

#### Real-time image reconstruction

A “real-time” (i.e., ungated before sorting into multiple time dimensions) image was first generated for motion identification:

3 $${\mathbf{X}}_{{{\text{rt}}}} = {\mathbf{U}}_{{{\mathbf{x}},{\text{rt}}}} {{\varvec{\Phi}}}_{{{\text{rt}}}}$$

where rows of ${{\varvec{\Phi}}}_{\text{rt}}$ correspond to the real-time temporal basis functions, and columns of ${\mathbf{U}}_{\mathbf{x},\text{rt}}$ correspond to the spatial basis functions.

The real-time temporal basis ${{\varvec{\Phi}}}_{\text{rt}}$ was estimated from the SVD of the training data ${\mathbf{d}}_{\text{tr}}$. Then the spatial coefficients ${\mathbf{U}}_{\mathbf{x},\text{rt}}$ were recovered by solving the least-squares optimization problem:

4 $${\hat{\mathbf{U}}}_{{{\mathbf{x}},{\text{rt}}}} = \arg\min_{{{\mathbf{U}}_{{{\text{rt}}}} }} \|{\mathbf{d}}_{{{\text{im}}}} - {\Omega }\left( {{\mathbf{EU}}_{{{\mathbf{x}},{\text{rt}}}} {{\varvec{\Phi}}}_{{{\text{rt}}}} } \right)\|_{2}^{2}$$

#### Respiratory and cardiac binning

Different time points of ${\mathbf{X}}_{\text{rt}}$ were assigned to multiple respiratory motion states (“respiratory bins”) and cardiac motion states (“cardiac bins”) based on the real-time images generated in Step 2. A modified k-means clustering method was used to automatically group the data into different bins, incorporating the predetermined low-rank T_1_ recovery model in ${\mathbf{U}}_{{\text{T}}_{1}}$ to address the contrast change of ${\mathbf{X}}_{\text{rt}}$ from T_1_ recovery [16].

#### Tensor formation and multidimensional reconstruction

After cardiac and respiratory binning, each readout time point was assigned three temporal indices: cardiac phase, respiratory phase, and T_1_ recovery index. A 4-way training data tensor ${\mathcal{D}}_{\text{tr}}$ with one k-space readout dimension and three temporal dimensions was recovered from the subspace training data ${\mathbf{d}}_{\text{tr}}$ by solving a Bloch-constrained low-rank tensor completion problem.

After ${\mathcal{D}}_{\text{tr}}$ is completed, the cardiac basis functions ${\mathbf{U}}_{\text{c}}$, the respiratory basis functions ${\mathbf{U}}_{\text{r}}$, and the core tensor $\mathcal{C}$ can be extracted from the HOSVD of ${\mathbf{D}}_{\text{tr}}$**,** fully determining ${\varvec{\Phi}}={\mathbf{C}}_{\left(1\right)}{\left({\mathbf{U}}_{\text{c}}\otimes {\mathbf{U}}_{\text{r}}\otimes {\mathbf{U}}_{{\text{T}}_{1}}\right)}^{\text{T}}$. The spatial coefficients ${\mathbf{U}}_{\mathbf{x}}$ can then be reconstructed by solving the problem in Eq. (2).

### Parameter fitting and image analysis

One specific respiratory phase and cardiac phase were selected for T_1_ and ECV map generation, corresponding to end-expiration and end-diastole respectively. A joint pre- and post-Gd T_1_ recovery model was also used in pixel-wise T_1_ fitting. By fitting this joint model (see Appendix), we can get the pre- and post-Gd T_1_ maps simultaneously.

#### Myocardial T_1_ maps

Parameter fitting was done using the joint pre- and post-Gd T_1_ recovery model in Eq. (11). ${T}_{1,\text{pre}},{T}_{1,\text{post}},\alpha ,B,$ and ${M}_{z}^{0}$ were fitted using the 832 inversion time images at the selected reparatory and cardiac phase. The pre- and post-Gd myocardial T_1_ maps were directly generated from the ${T}_{1,\text{pre}}$ and ${T}_{1,\text{post}}$ fitting results.

#### Blood T_1_ fitting

First, the pixels to be used for blood T_1_ fitting were automatically selected based on the thresholding of the fitted ${M}_{z}^{0}$ map. Then, the average T_1_ recovery curve from these pixels was calculated, weighted by the ${M}_{z}^{0}$ map. Finally, parameters were fitted to the average blood T_1_ recovery curves using the model in Eq. (11) without Look–Locker correction (i.e., with ${M}_{n}\left({M}_{z}^{0},{T}_{1},\alpha =0,B\right)$) to better model the inflow of unexcited blood spins into the imaging slice [17]. The fitted ${T}_{1,\text{pre}}$ and ${T}_{1,\text{post}}$ values were then used as the pre- and post-Gd blood T_1_ values.

#### ECV calculation

The ECV map was generated according to the following equations:

5 $${\text{ECV}} = \left( {1 - {\text{HCT}}} \right) \cdot \frac{{{\Delta }R_{{1,{\text{myo}}}} }}{{{\Delta }R_{{1,{\text{blo}}}} }} \times 100\%$$

6 $${\Delta }R_{1} = R_{{1,{\text{post}}}} - R_{{1,{\text{pre}}}} = \frac{1}{{T_{{1,{\text{post}}}} }} - \frac{1}{{T_{{1,{\text{pre}}}} }}$$

in which ${\Delta }{R}_{1,myo}$ is the pixel-wise ${R}_{1}$ changes, and $\Delta {R}_{1,\text{blo}}$ is the change of blood ${R}_{1}$, and HCT is the hematocrit value. All statistical analyses were performed for septal ECV, i.e., where $\Delta {R}_{1,\text{myo}}$ was calculated as the mean value within the septal myocardium. All image reconstruction and curve fitting was done in MATLAB 2018a (MathWorks, Natick, Massachusetts, USA).

### Phantom validation

The accuracy of T_1_ fitting of the pre- or post-Gd T_1_ Multitasking sequence was tested in an 8-vial water phantoms with GdCl_3_ concentrations of 0, 10, 20, 33, 50, 80, 120 and 200 umol/kg. T_1_ maps generated by the Bruker built-in RARE-VTR method was used as the reference. The imaging parameters of RARE-VTR were: TE = 19.5 ms, TR array = 8000, 4000, 1500, 800, 400, 200 and 120 ms, RARE factor = 4, matrix size = 128 × 128, FOV = 40 × 40 mm^2^, and total scan time = 6 min 1 s.

### Histological analysis

Masson's trichrome staining was used to measure the extent of fibrosis. Mid-ventricular heart tissues were sectioned and stained following the manufacturer’s protocol (Sigma-Aldrich, St. Louis, Missouri, USA). Quantitative histological analysis was done with ImageJ 1.52a (National Institutes of Health; http://imagej.nih.gov/ij). In each section, the extent of myocardial fibrosis was quantified by the percentage of total fibrosis area, which was calculated as the number of blue-stained pixels divided by the total number of pixels in the ventricular area (excluded: intramural vascular structures, perivascular collagen, endocardium, and LV trabeculae) [20, 30]. For each rat, the average percentage from five different sections was reported.

### Statistical analysis

Welch’s t-test was performed to compare ECV values between the control group and HFpEF group. The Pearson coefficient was measured to evaluate the correlation between ECV values and quantitative fibrosis percentages. A two-tailed value of *P* < 0.05 was considered to be statistically significant. Statistical graphs were generated using GraphPad Prism 8 (GraphPad Software, San Diego, California, USA) and Excel (Microsoft Corporation, Redmond, Washington, USA).

## Results

**Fig. 3:**
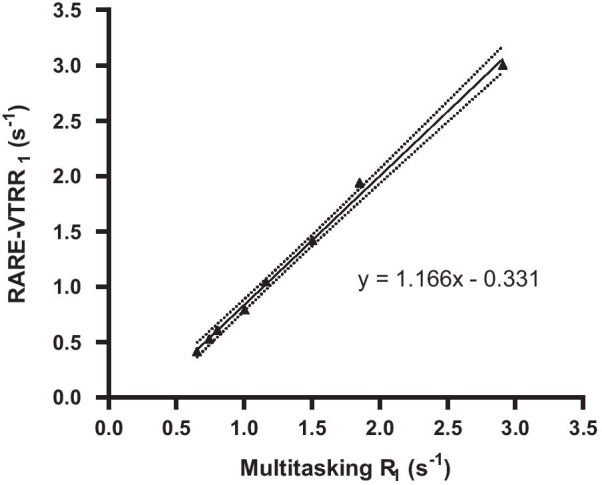
Linear regression of Multitasking and RARE-VTR R_1_ values. Linear regression showed a strong linear relationship in R_1_ between Multitasking and RARE-VTR measurements (*R* = 0.99, *P* < 0.001). The dotted line shows the 95% confidence bonds

The reference T_1_ of the phantoms measured by RARE-VTR ranged from 333 to 2433 ms, with a corresponding R_1_ range from 0.41 to 3.00 s^−1^. Figure 3 shows the linear regression result of R_1_ values measured by Multitasking and RARE-VTR, in which a strong linear relationship can be found (*R* = 0.9983, *P* < 0.0001).

**Fig. 4:**
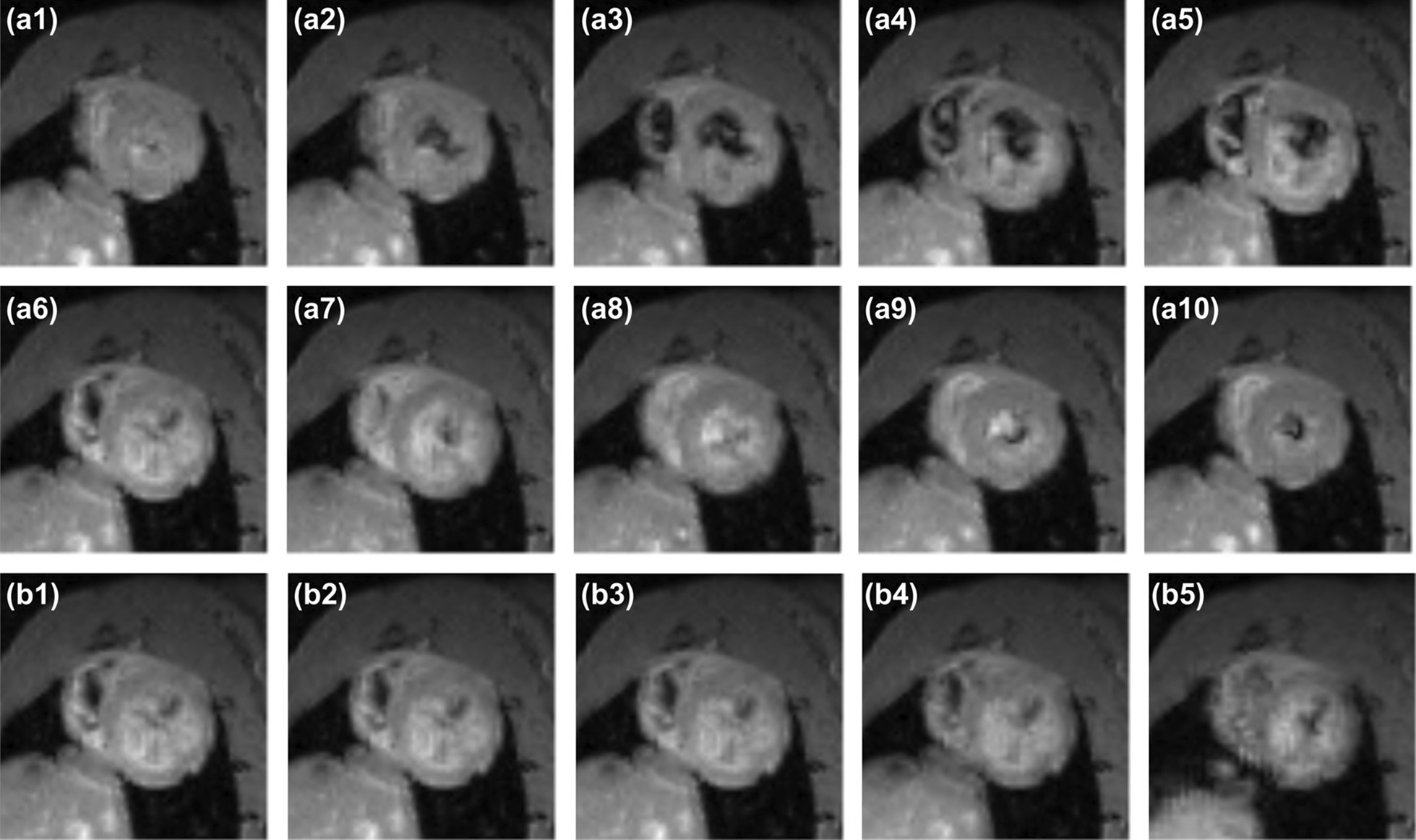
Images of different cardiac and respiratory bins. Representative images of **a** ten different cardiac bins (a1–a10), and **b** five different respiratory bins (b1–b5) at the beginning of an inversion recovery preparation pulse. Movies are available in Additional file 1, showing the dynamic images from (1) different respiratory bins, (2) different cardiac bins, (3) native T_1_ recovery process, and (4) post-Gd T_1_ recovery process

Five respiratory bins and ten cardiac bins were used for respiratory and cardiac binning, corresponding to a cardiac temporal resolution of 20 ms for a heart rate of ~ 300 bpm. Figure 4 shows the images of the heart from a representative healthy control rat right before the IR preparation pulse, generated from different cardiac and respiratory phases, i.e. ${\left.\mathcal{A}\left(\text{x},{n}_{\text{c}},{n}_{\text{r}},{n}_{{\text{T}}_{1}}\right)\right|}_{{ n}_{\text{c}}=1:10, {n}_{\text{r}}=1, {n}_{{\text{T}}_{1}}=N=416}$ (Fig. 4a) and ${\left.\mathcal{A}\left(\mathbf{x},{n}_{\text{c}},{n}_{\text{r}},{n}_{{\text{T}}_{1}}\right)\right|}_{{ n}_{\text{c}}=6, {n}_{\text{r}}=1:5, {n}_{{\text{T}}_{1}}=N=416}$ (Fig. 4b). The diastole (Fig. 4a6) and the systole (Fig. 4a1), or the end-expiration phase (Fig. 4b1) and the end-inspiration phase (Fig. 4b5) can be clearly differentiated in the figure. Movies showing the dynamic images from (1) different respiratory bins, (2) different cardiac bins, (3) native T_1_ recovery process, and (4) post-Gd T_1_ recovery process are also available in Additional file 1.

**Fig. 5:**
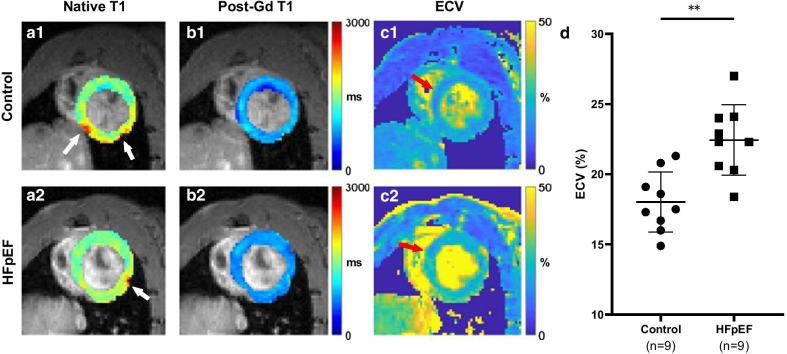
Representative T_1_ and Extracellular volume fraction (ECV) maps (local) from the control and heart failure with preserved ejection fraction (HFpEF) group. **a**–**c** Representative T_1_ and ECV maps from the control group (1) and HFpEF group (2). Myocardial R_1_ changes for the final ECV analysis were calculated within septal myocardium, as indicated by red arrows in **c**. **d** ECV values were significantly higher in HFpEF group (22.43% ± 2.51%), compared with the control group (18.02% ± 2.13%), *P* = 0.0010

Figure 5a, b shows representative native and post-Gd T_1_ maps from the control group and heart failure with preserved ejection fractino (HFpEF) group. T_1_ maps were smooth and homogeneous within the left ventricular (LV) myocardium, except for areas in the inferior and lateral walls with imperfect fitting results (as indicated by white arrows in Fig. 5a1, a2). The native myocardial T_1_ values (mean ± SD, in ms) were 1662 ± 152 in the HFpEF group (vs 1534 ± 151 in the control group; *P* = 0.09). Figure 5c shows the corresponding ECV maps. In this study, signal from the aorta rather than the LV was usually selected for blood T_1_ fitting, because of the signal loss resulting from LV inflow in this single slice setup; and the septal myocardium areas selected for $\Delta {R}_{1,\text{myo}}$ calculation were indicated by red arrows. Welch’s t-test showed that ECV was significantly higher in the HFpEF group (22.4% ± 2.5% vs. 18.0% ± 2.1%; *P* = 0.0010, Fig. 5d).

**Fig. 6:**
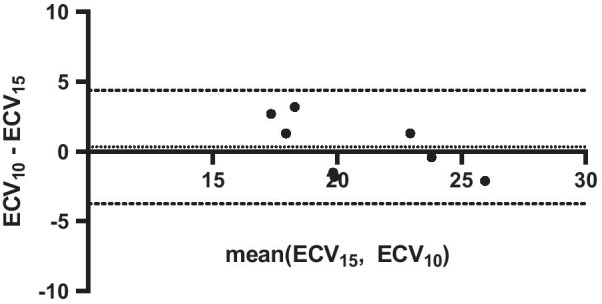
The Bland–Altman plot evaluating the repeatability of ECV measurement. The dotted and dashed lines indicate the mean bias and the 95% limit of agreement, respectively

In eight rats (4 control + 4 HFpEF), an additional post-Gd T_1_ Multitasking sequence was run 10 min after the Gd injection, with identical imaging parameters. ECVs were also calculated using this separate post-Gd T_1_ measurement. Figure 6 shows the Bland–Altman plot comparing the ECVs measured from the 15-min post-Gd T_1_ (ECV_15_) and the 10-min post-Gd T_1_ (ECV_10_). The interclass correlation coefficient (ICC) was 0.817. The root-mean-square within-subject standard deviation was 1.4, yielding a coefficient of variation of 6.7%. There was no significant difference between ECV_15_ and ECV_10_ (*P* = 0.66).

**Fig. 7:**
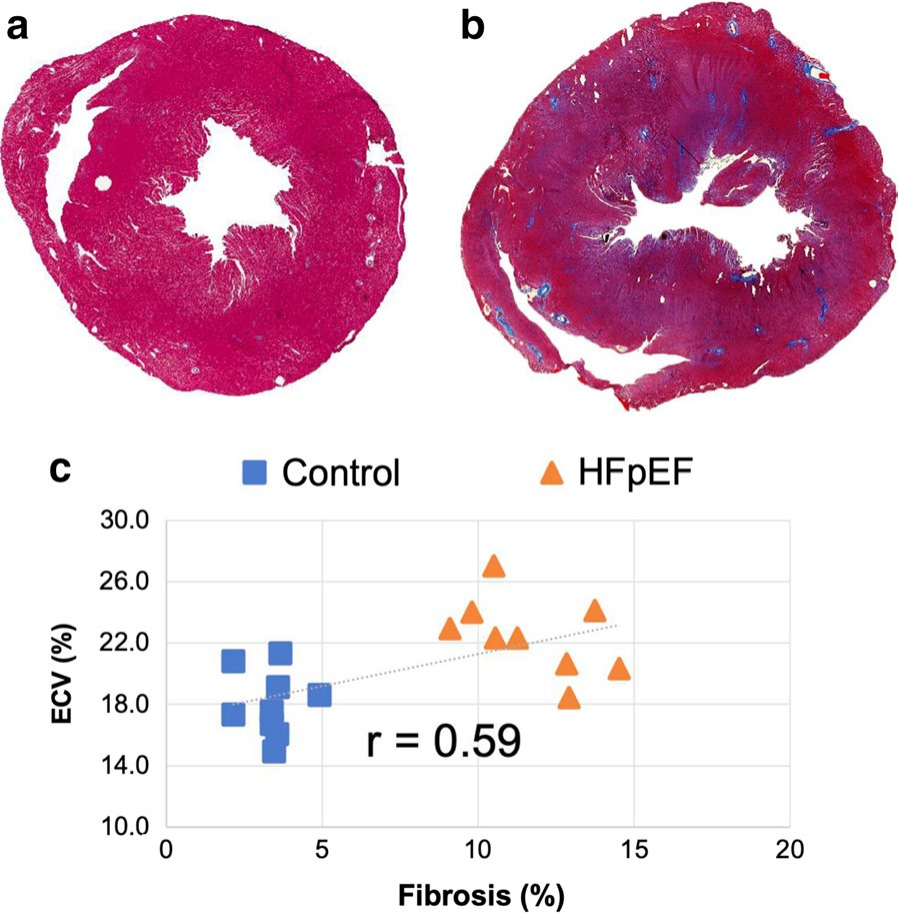
Representative histological sections and ECV(%)-fibrosis(%) scatterplot. **a**, **b** Representative Masson trichrome-stained sections of control group and HFpEF group. The extent of fibrosis was significantly higher in HFpEF hearts (11.7% ± 1.9%) than in control hearts (3.4% ± 0.8%), *P* < 0.001. **c** ECV values and the extent of fibrosis showed a moderate correlation: *R* = 0.59, *P* = 0.0098

Figure 7a, b shows representative Masson trichrome-stained sections of a HFpEF and control rat. The myocardial fibrosis can be clearly seen as the diffused dark blue areas in Fig. 7b. The extent of fibrosis significantly increased in HFpEF hearts (11.7% ± 1.9% vs 3.4% ± 0.8%, *P* < 0.001) Fig. 7c shows the relationship between the ECV value and the extent of fibrosis, in which a moderate correlation can be found (*R* = 0.59, *P* = 0.0098).

## Discussion

We developed a novel ECV Multitasking protocol which can map ECV at high heart rates without ECG triggering or respiratory navigation. To test its feasibility to characterize diffuse myocardial fibrosis, a HFpEF rat model was chosen in this study. Although ECV has not previously been measured in a HFpEF rat model, there has been increasing clinical interest in T_1_ and ECV characterization of patients with HFpEF in recent years. Several publications showed elevated ECV in HFpEF patients versus control subjects. Su et al. reported that patients with HFpEF had elevated ECV compared with control subjects (28.9% vs. 27.9%, *P* = 0.006) [6]. Rommel et al. reported similar results (32.9% vs. 28.9%, *P* < 0.01) [5], and Mordi et al. also confirmed this finding (35.9% vs. 27.0%, *P* < 0.001) [4]. A modified Look-Locker inversion recovery (MOLLI) sequence was used to perform T_1_ mapping in all of these studies. In the present study, ECV characterization was performed for the first time in a HFpEF rat model. The elevated ECV we found with HFpEF (22.4% vs. 18.0%, *P* = 0.001) is consistent with previous human studies. Though so far only tested in the DSS rat model for HFpEF diagnosis [18, 19], this newly developed technique can easily be extended to other small animal applications. More importantly, the non-invasive quantitative imaging protocol not only provides a diagnostic tool, but also a method for longitudinal therapy monitoring of the same subject [31].

In the control group, native T_1_ values were 1534 ± 151 ms and ECV values were 18.0% ± 2.1% (both calculated from the septal area). Previous rodent studies have reported variable myocardial T_1_ values, depending on field strength and T_1_ encoding schemes, both of which are known to affect T_1_ estimates. At 7.0 T, T_1_ values were reported as 1620 ms (Vandsburger et al.) and 1638 ms (Zhang et al.) [10, 32]. At 9.4 T, reported T_1_ values ranged from ~ 1200 ms to 1764 ms (Kim et al. Li et al. and Coolen et al.) [7, 33, 34]. ECV, a physiological quantity in principle unaffected by field strength or T_1_ encoding scheme, allows more direct comparison. In healthy rats, myocardial ECV was previously reported as 16% [35], 18% [36], 17.2% [9], and 15.5% [34], comparable with the 18.0% value measured in our study. A direct comparison to these previous methods on the same scanner and in the same subjects was not performed in the present study, but would be a useful future validation step.

One major technical novelty of this work compared with previous CMR Multitasking work is the joint reconstruction of pre- and post-Gd data and the paired modeling of the pre- and post-Gd T_1_ recovery. This has two primary benefits. First, it allows the respiratory and cardiac binning to be done in the concatenated pre- and post-Gd data. In this way, the data with a similar motion state—no matter whether from pre-Gd or post-Gd—will be clustered into exactly the same respiratory or cardiac bin, removing the need for respiratory co-registration between pre- and post-Gd T_1_ maps. Second, it can improve the robustness of T_1_ fitting by constraining pre- and post-Gd images to share the same thermal equilibrium magnetization ${M}_{z}^{0}$, FLASH flip angle $\alpha$ and inversion efficiency $B$, exploiting internal a priori knowledge and reducing the total number of fitting parameters from 8 to 5.

The phantom study found that the Multitasking based T_1_ fitting results showed a strong linear correlation with the RARE-VTR reference. If calibrated using the linear correlation (${\stackrel{\sim }{R}}_{1}=LC\left({R}_{1}\right)=1.166{R}_{1}-0.331$), the relative R_1_ difference between Multitasking method and RARE-VTR reference would be 2.7% ± 3.6%. Note that ECV measurements are insensitive to linear transformation: the ECV value is calculated by the ratio of R_1_ changes of myocardial and blood pool, so both the intercept (− 0.331) and slope (1.166) of the linear transformation $LC$ will be canceled, leaving the final ECV quantification result unchanged.

Ten bins were used to separate different cardiac phases in the current protocol for CMR ECV Multitasking reconstruction, corresponding to a cardiac temporal resolution of 20 ms for a heart rate of 300 bpm. As shown in Fig. 2, the diastolic and systolic phases can be differentiated at this temporal resolution. However, for higher heart rates, such as those in mice (around 450 bpm), more cardiac bins may be required, which may have an impact on SNR and ECV homogeneity. A nearly-significant negative correlation (*R* = − 0.44, *P* = 0.06) was found between the septal SNR of the ECV map and the heart rate (the scatterplot can be found in Additional file 1). This may be an effect of increased intra-bin motion at higher heart rates. More cardiac bins can possibly be achieved by shortening the echo spacing, or by acquiring k-space training line and imaging lines in separate echoes after each FLASH flip angle [37].

In this work, blood T_1_ was extracted by fitting the weighted average T_1_ recovery curve of the selected blood pool. The FLASH flip angle $\alpha$ in Eq. (8) was fixed as 0 to remove Look-Locker correction and better model the inflow of unexcited blood spins. This approach is particular to 2D slice-selective excitation, for which blood spins flow through the slice quickly enough that Look-Locker correction is not required. For a volumetric 3D variation of our method, the blood spins would be driven towards steady-state, and the Look-Locker correction could be retained in blood.

T_1_ inhomogeneity was present in the inferior and lateral walls due to potential B_1_ issues at high B_0_ field, as visible in Fig. 4. In diffuse myocardial disease, it does not affect the ECV calculation from septal area. However, it will affect the accuracy of the ECV map in the inferior and lateral walls. This may reduce the reliability of the method in the diagnosis of focal myocardial disease, such as focal myocardial infarction. Further sequence improvements, such as a dual-flip-angle acquisition [38], should be made to address this problem.

The method described here did not include bulk motion compensation, as anesthesia and additional immobilization setup did not result in any apparent bulk motion. However, bulk motion compensation is compatible with the CMR Multitasking framework, as described in [39], where a translational inter-bin registration to k-space data was applied prior to joint reconstruction. Similar motion compensation could be incorporated into the proposed method if used with different experimental setups more susceptible to bulk motion.

Currently, a total of 85 IR preparation pulses were applied in each T_1_ Multitasking module, resulting in a single-slice scan of just over 4 min. However, the minimum required scan time was not systematically explored, so further shortening of the scan time may yet be possible.

Future work will include optimizing the number of cardiac bins, expanding spatial coverage with 3D volumetric imaging, as well as addressing B_1_ and B_0_ issues affecting T_1_ homogeneity in the inferior and lateral walls. Further imaging tests will also include using ECV Multitasking for longitudinal therapy monitoring, such as the cardiosphere-derived cell (CDC) treatment of HFpEF [31].

## Conclusions

We developed an ECG-less, free-breathing CMR Multitasking ECV mapping method at high heart rates. The pre- and post-Gd data were concatenated and reconstructed using a joint T_1_ recovery model to generate ECV maps. Elevated ECV found in the HFpEF group agrees well with previous human studies and shows a moderate correlation with the histological data. This technique can serve as a viable CMR imaging tool for myocardial tissue characterization in small animal models.

## Supplementary Information

**Additional file 1: Page 1.** The four videos show reconstructed results of (1) steady-state images from five respiratory bins, (2) steady-state images from ten cardiac bins, (3) native inversion recovery process, and (4) post-Gd inversion recovery, respectively. **Page 2:** The scatterplot between the SNR of the ECV map and the heart rate. The SNR was measured as mean/std of the ECV map within the septal area. A nearly-significant negative correlation (R = – 0.44, P = 0.06) can be found between the SNR and the heart rate.

## Data Availability

The datasets acquired and/or analyzed during the current study are not publicly available but are available from the corresponding author on reasonable request.

## Appendix: T_1_ recovery modelling

The z-magnetization within a single recovery period between two adjacent inversion recovery (IR) pulses can be described as

7 $${M}_{n}={M}_{\text{ss}}+\left(B{M}_{N+1}-{M}_{\text{ss}}\right)\cdot {E}^{n-1}$$

8 $$E={e}^{-\frac{TR}{{T}_{1}}}\text{cos}\alpha ,$$

9 $${M}_{N+1}={M}_{ss}\frac{1-{E}^{N}}{1-B{E}^{N}},$$

where $n$ is the readout index within each recovery period, $n=1, 2, \ldots ,N$; ${M}_{ss}=\frac{1-{e}^{-TR/{T}_{1}}}{1-{e}^{-\mathit{TR}/{T}_{1}}\mathit{cos}\alpha }{M}_{z}^{0}$ is the z-magnetization at true steady-state ($t\to \infty$); ${M}_{N+1}$ is the actual final z-magnetization in each recovery period incorporating incomplete relaxation; and $B\in \left[-\text{1,0}\right]$ represents the inversion efficiency of the IR pulse. Our pulse sequence used $N=416$, TR = 7 ms; $\alpha$ was prescribed as 5° but was not assumed to be perfectly homogenous during Bloch simulations and during fitting.

Combining Eqs. 7, 8, 9 yields the combined z-magnetization equation

10 $${M}_{n}\left({M}_{z}^{0},{T}_{1},\alpha ,B\right)={M}_{ss}\left({M}_{z}^{0},{T}_{1},\alpha \right)\cdot \left[1+\left(B\frac{1-{e}^{-\frac{TR}{{T}_{1}}}\cos\alpha }{1-B{e}^{-\frac{TR}{{T}_{1}}}\cos\alpha }-1\right)\cdot {\left({e}^{-\frac{TR}{{T}_{1}}}\cos\alpha \right)}^{n-1}\right]$$

Assuming that the B_1_ field does not change between pre- and post-contrast acquisitions, the unknown actual FLASH flip angle $\alpha$ and inversion efficiency $B$ can be assumed to be constant for each pixel. Therefore the paired signal model concatenating the pre- and post-Gd signal evolution is:

11 $$S\left({M}_{z}^{0},{T}_{1,\text{pre}},{T}_{1,\text{post}},\alpha ,B\right)=\left\{{M}_{1:N}\left({M}_{z}^{0},{T}_{1,\text{pre}},\alpha ,B\right), {M}_{1:N}\left({M}_{z}^{0},{T}_{1,\text{post}},\alpha ,B\right)\right\}\cdot \sin\alpha$$

## Abbreviations

CDC: Cardiosphere-derived cell
CMR: Cardiovascular magnetic resonance
DSS: Dahl salt-sensitive
ECG: Electrocardiogram
ECV: Extracellular volume fraction
FLASH: Fast low angle shot
HCT: Hematocrit
HFpEF: Heart failure with preserved ejection fraction
HOSVD: High-order singular value decomposition
HS: High salt
IR: Inversion recovery
LV: Left ventricle/left ventricular
MOLLI: Modified Look-Locker inversion recovery
NS: Normal salt
RARE: Rapid acquisition with relaxation enhancement
SALLI: Small animal Look-Locker inversion recovery
SNR: Signal-to-noise ratio
SVD: Singular value decomposition
VFA: Variable flip angle
