# Publisher Correction to: Electrocardiogram-less, free-breathing myocardial extracellular volume fraction mapping in small animals at high heart rates using motion-resolved cardiovascular magnetic resonance multitasking: a feasibility study in a heart failure with preserved ejection fraction rat model

**DOI:** 10.1186/s12968-021-00738-z

**Published:** 2021-03-22

**Authors:** Pei Han, Rui Zhang, Shawn Wagner, Yibin Xie, Eugenio Cingolani, Eduardo Marban, Anthony G. Christodoulou, Debiao Li

**Affiliations:** 1 Biomedical Imaging Research Institute, Cedars-Sinai Medical Center, Los Angeles, CA USA; 2 Department of Bioengineering, University of California, Los Angeles, Los Angeles, CA USA; 3 Smidt Heart Institute, Cedars-Sinai Medical Center, Los Angeles, CA USA; 4 Department of Cardiology, Xinhua Hospital Affiliated to Shanghai Jiaotong University School of Medicine, Shanghai, China

## Correction to: J Cardiovasc Magn Reson 23:8 (2021) https://doi.org/10.1186/s12968-020-00699-9

Two errors have been corrected in the original article [1] following the publication:

1. The order of affiliations has been corrected as per the corresponding authorship.
2. The article title read “reesonance” this has been corrected to “resonance”.

These errors have been updated in this correction article and the original article. The publisher apologizes to the authors and readers for the inconvenience.
