# Supplementary figures and images for: Electrocardiogram-less, free-breathing myocardial extracellular volume fraction mapping in small animals at high heart rates using motion-resolved cardiovascular magnetic reesonance multitasking: a feasibility study in a heart failure with preserved ejection fraction rat model
Source: J Cardiovasc Magn Reson. 2021 Feb 11;23:8. doi: 10.1186/s12968-020-00699-9 (PMC7877086; doi:10.1186/s12968-020-00699-9)

## Slide 1
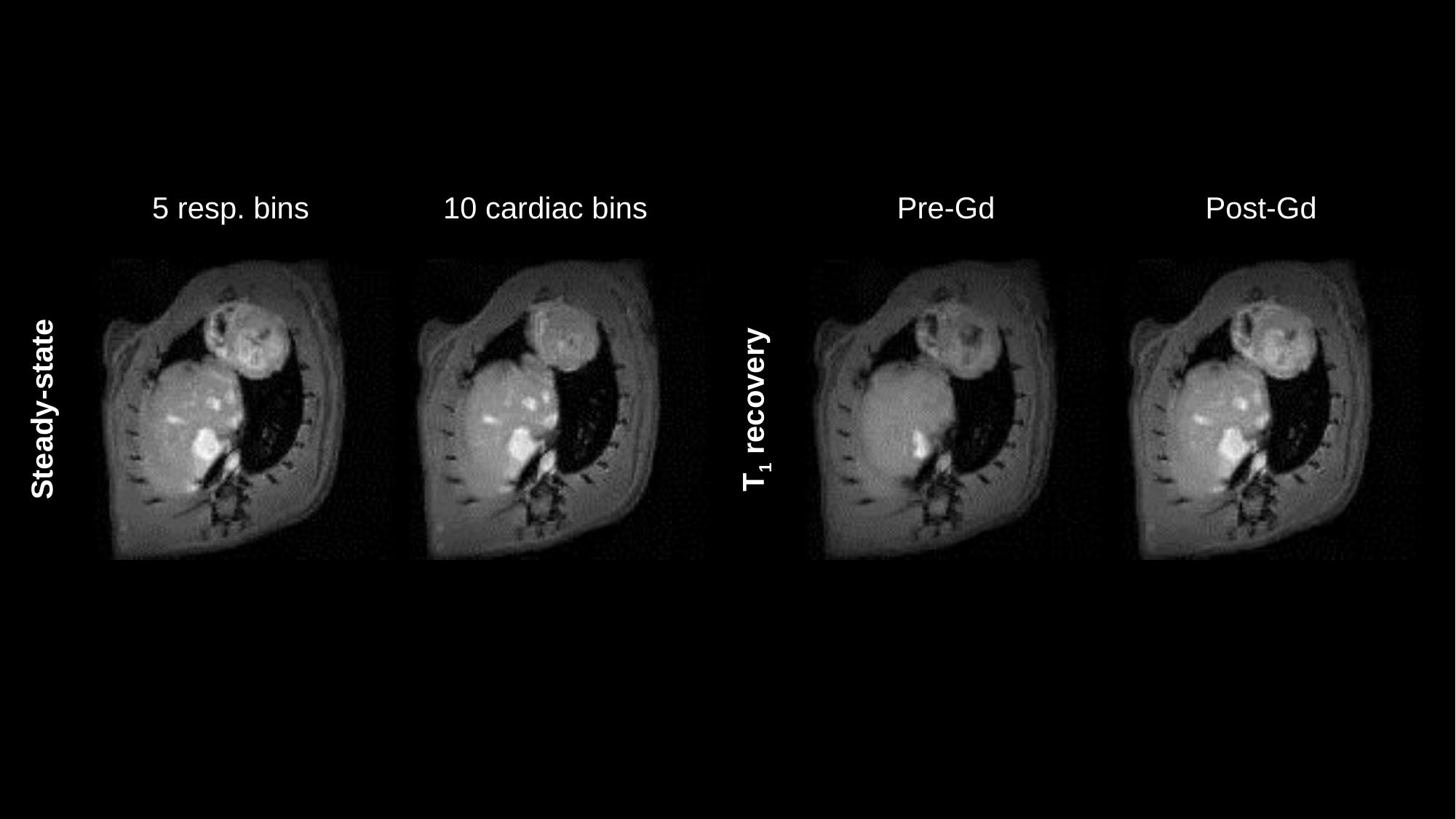

T1 recovery
Steady-state
5 resp. bins
10 cardiac bins
Pre-Gd
Post-Gd

## Slide 2
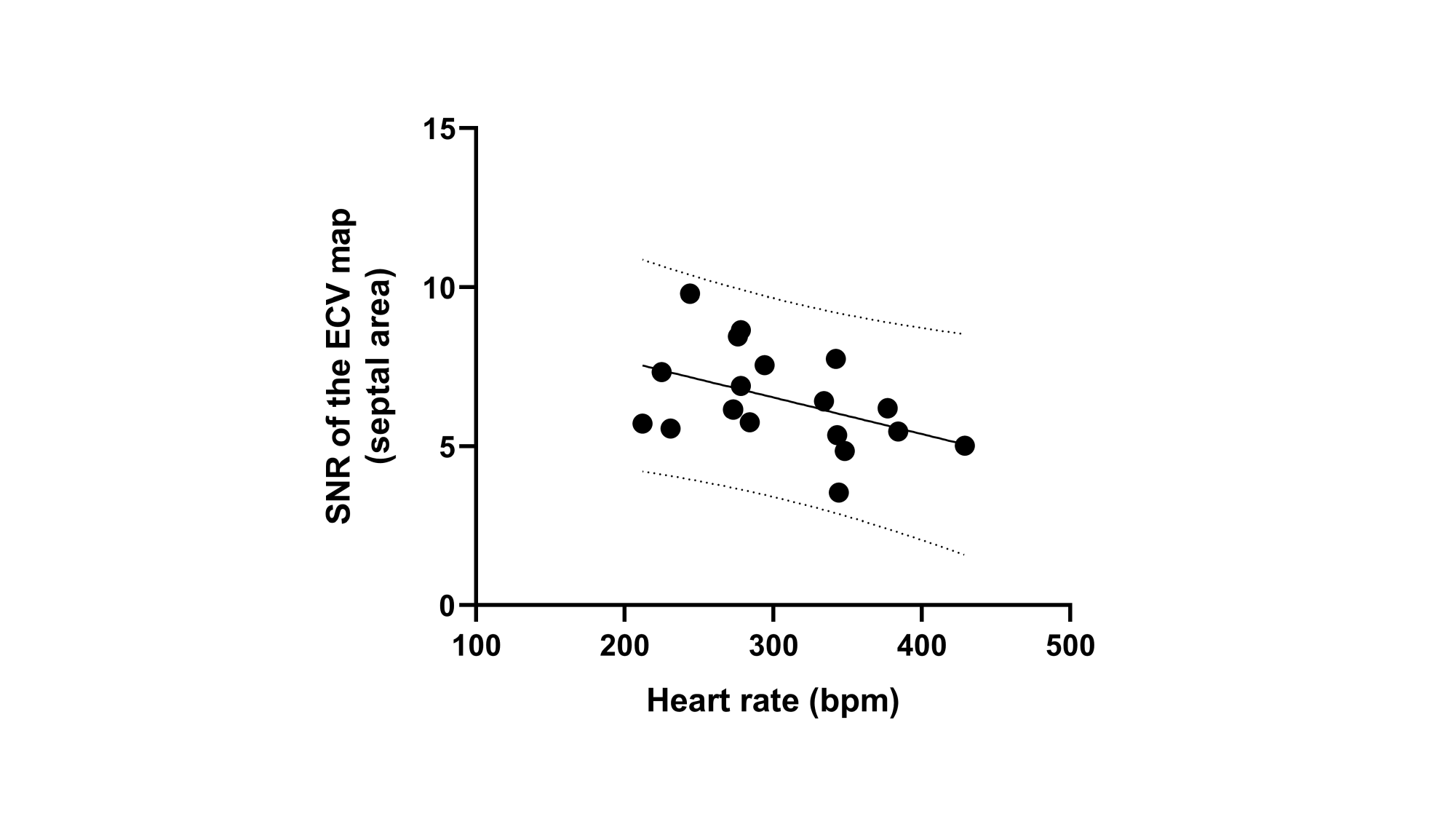

Supplement: Supplementary file 1 — Additional file 1: Page 1. The four videos show reconstructed results of (1) steady-state images from five respiratory bins, (2) steady-state images from ten cardiac bins, (3) native inversion recovery process, and (4) post-Gd inversion recovery, respectively. Page 2: The scatterplot between the SNR of the ECV map and the heart rate. The SNR was measured as mean/std of the ECV map within the septal area. A nearly-significant negative correlation (R = – 0.44, P = 0.06) can be found between the SNR and the heart rate. [file 12968_2020_699_MOESM1_ESM.pptx]
